# Case Report: a 28-year-old female with anomalous origin of the left coronary artery from the pulmonary artery syndrome presented as atrial fibrillation and pulmonary hypertension

**DOI:** 10.3389/fcvm.2025.1573539

**Published:** 2025-05-12

**Authors:** Keyue Sun, Fan Guo, Xiqi Xu, Yongtai Liu

**Affiliations:** Department of Cardiology, Peking Union Medical College Hospital, Peking Union Medical College & Chinese Academy of Medical Sciences, Beijing, China

**Keywords:** ALCAPA, anomalous origin of the left coronary artery from the pulmonary artery, atrial fibrillation, pulmonary hypertension, case report

## Abstract

Anomalous origin of the left coronary artery from the pulmonary artery (ALCAPA) typically manifests in the first weeks of life. We report a 28-year-old woman with atrial fibrillation and pulmonary hypertension which were later found to be associated with ALCAPA syndrome. Despite a history free of traditional cardiovascular risk factors, her symptoms included exercise intolerance, palpitations, and an ischemic stroke. Echocardiography and further examination revealed pulmonary artery origin of the left coronary artery and extensive collateral formation between the left and right coronary arteries, contributing to her symptoms.

## Introduction

Anomalous origin of the left coronary artery from the pulmonary artery (ALCAPA) syndrome, also known as Bland-White-Garland syndrome, is a rare congenital anomaly affecting 1 in 300,000 live births (1). If untreated, ALCAPA has a high mortality rate, with most undiagnosed cases dying within the first year of life from myocardial ischemia or arrhythmia (2). If there is adequate coronary collateral between the right coronary artery (RCA) and the left coronary artery (LCA), symptoms of this anomaly may not appear until adulthood (3). However, the occurrence of ALCAPA in adults is exceedingly rare, and specific incidence data for this demographic are lacking because such cases are seldom reported. This case report describes a 28-year-old woman who presented with atrial fibrillation and pulmonary hypertension, symptoms that were later attributed to ALCAPA.

## Case description

**Figure 1:**
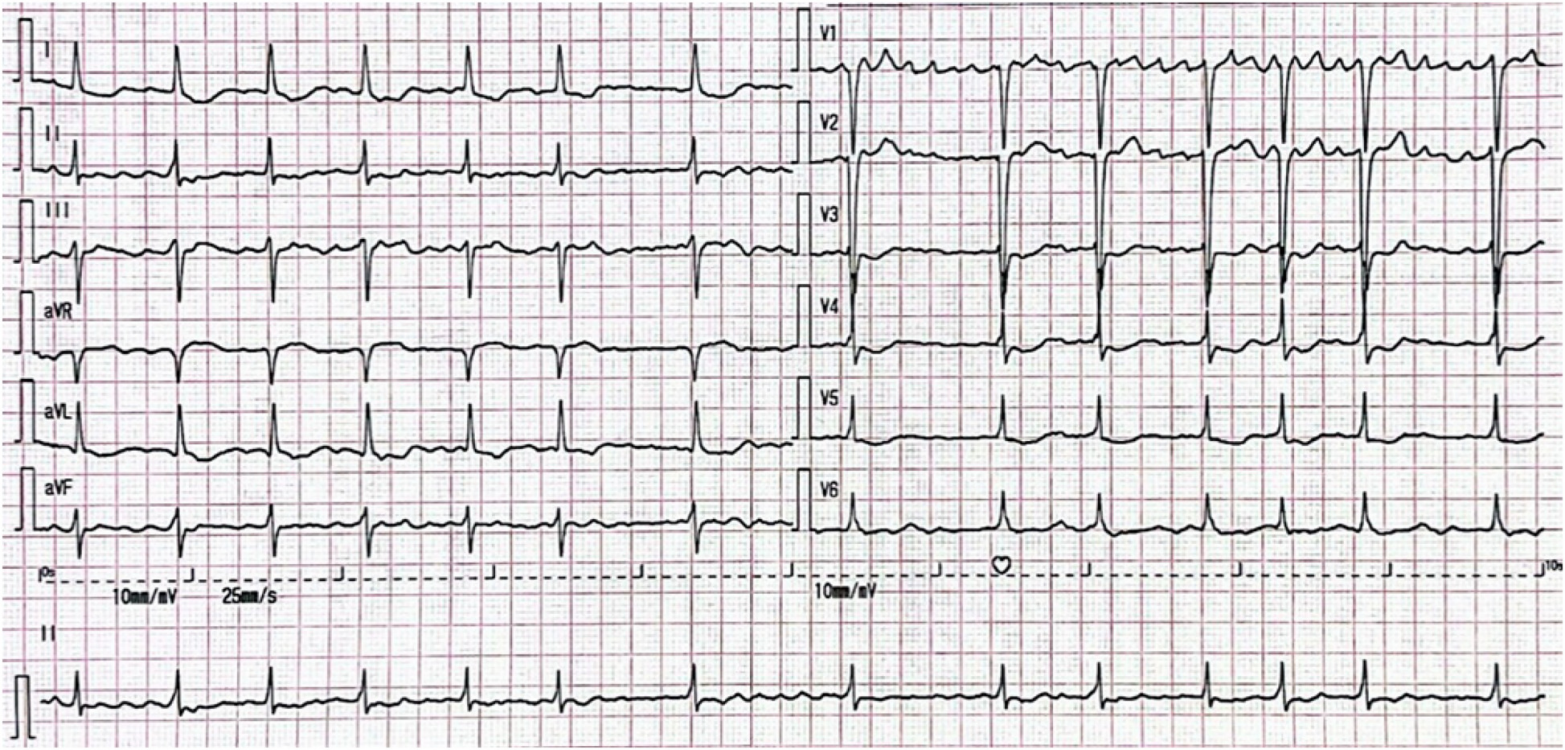
ECG. The ECG shows atrial fibrillation with a ventricular rate of 84 bpm, ST-segment depression in leads I, aVL and V4–V6, and poor R-wave progression in leads V1–V3.

**Figure 2:**
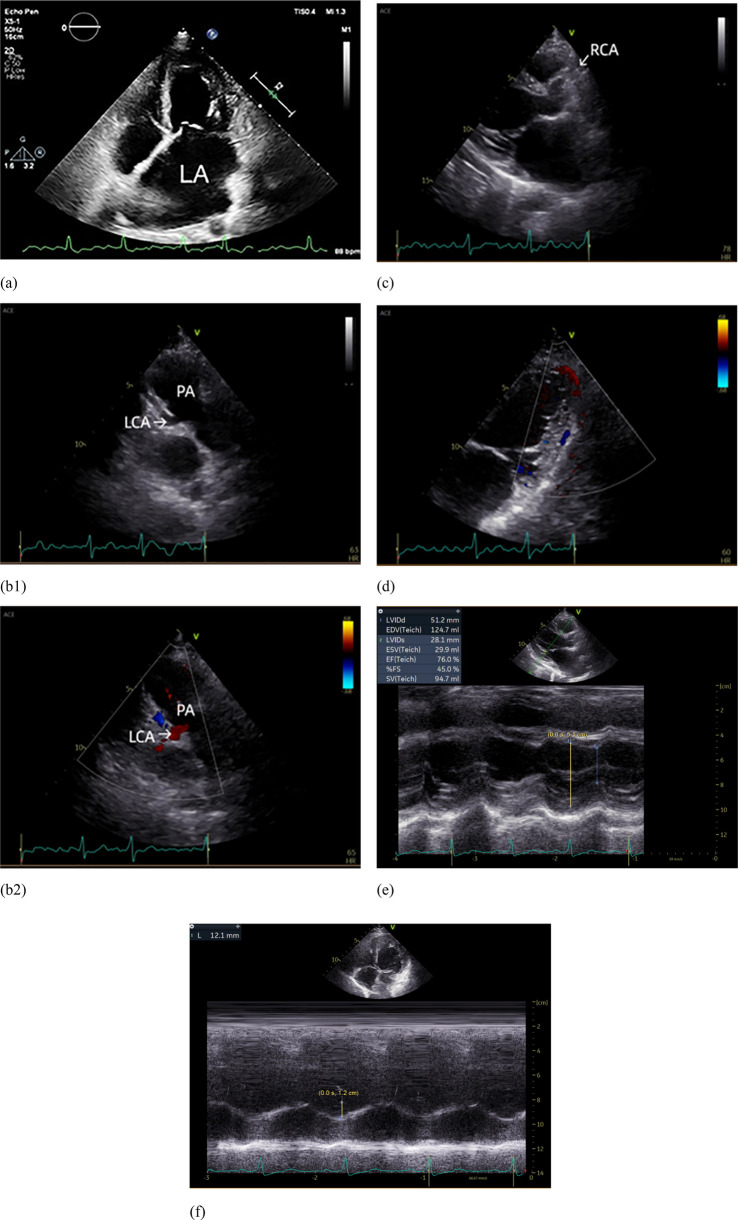
Echocardiograph. **(a)** The left atrium was markedly enlarged; **(b1)** The left coronary artery (LCA) originates from the pulmonary artery (PA); **(b2)** Blood flow from LCA to PA; **(c)** The right coronary artery (RCA) originates from the right coronary sinus and is dilated. **(d)** Intramyocardial visualization of blood flow signals between the LCA and RCA. **(e)** Dilatation of the left ventricle. **(f)** Reduced tricuspid annular plane systolic excursion (TAPSE).

**Figure 3:**
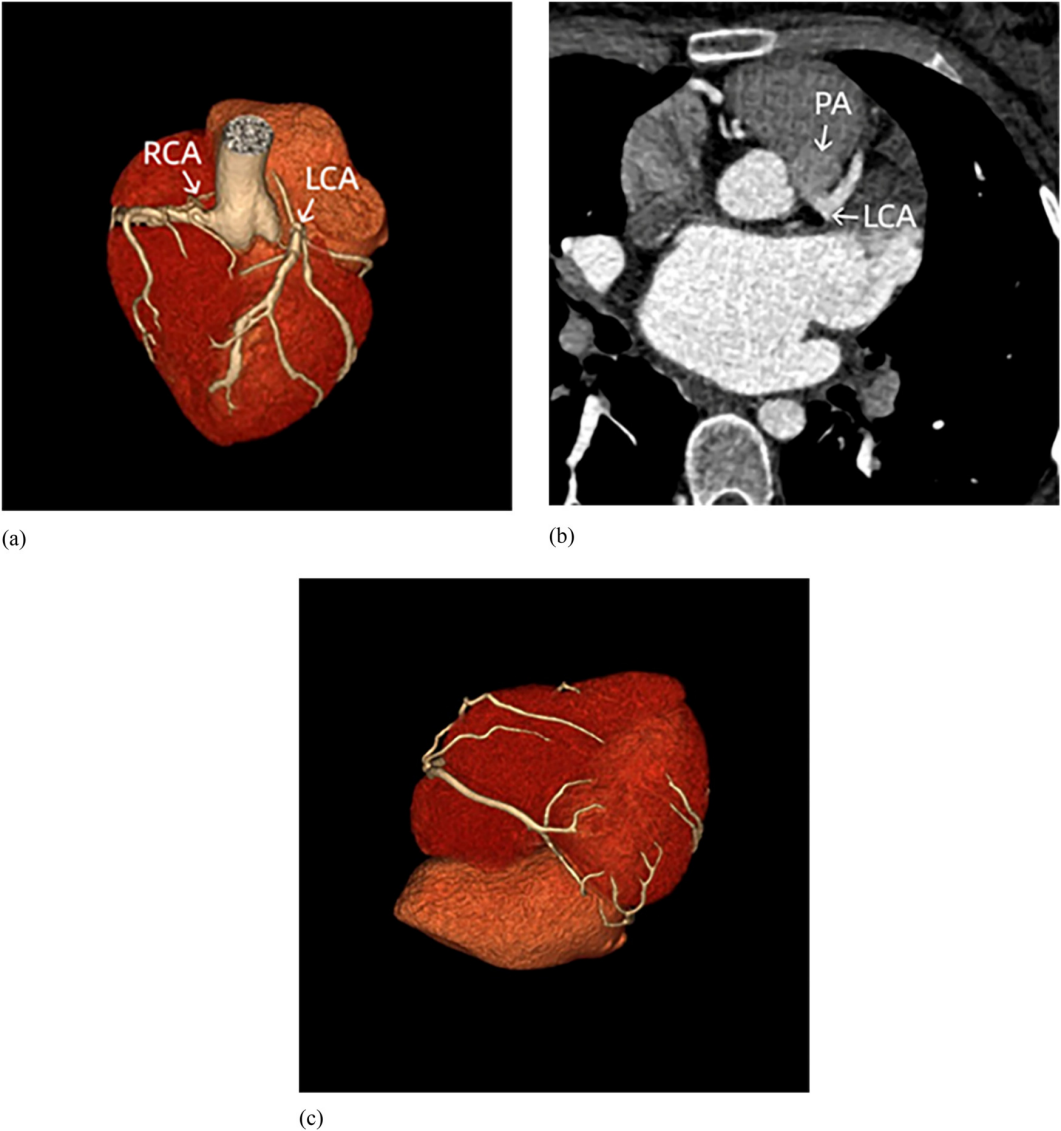
Coronary CT. **(a)** The right coronary artery (RCA) originates from the aortic root, while the left coronary artery (LCA) originates from the pulmonary artery (without pulmonary artery reconstruction). **(b)** The LCA originates from the pulmonary artery (PA). **(c)** Extensive communications exist between LCA and RCA.

**Figure 4:**
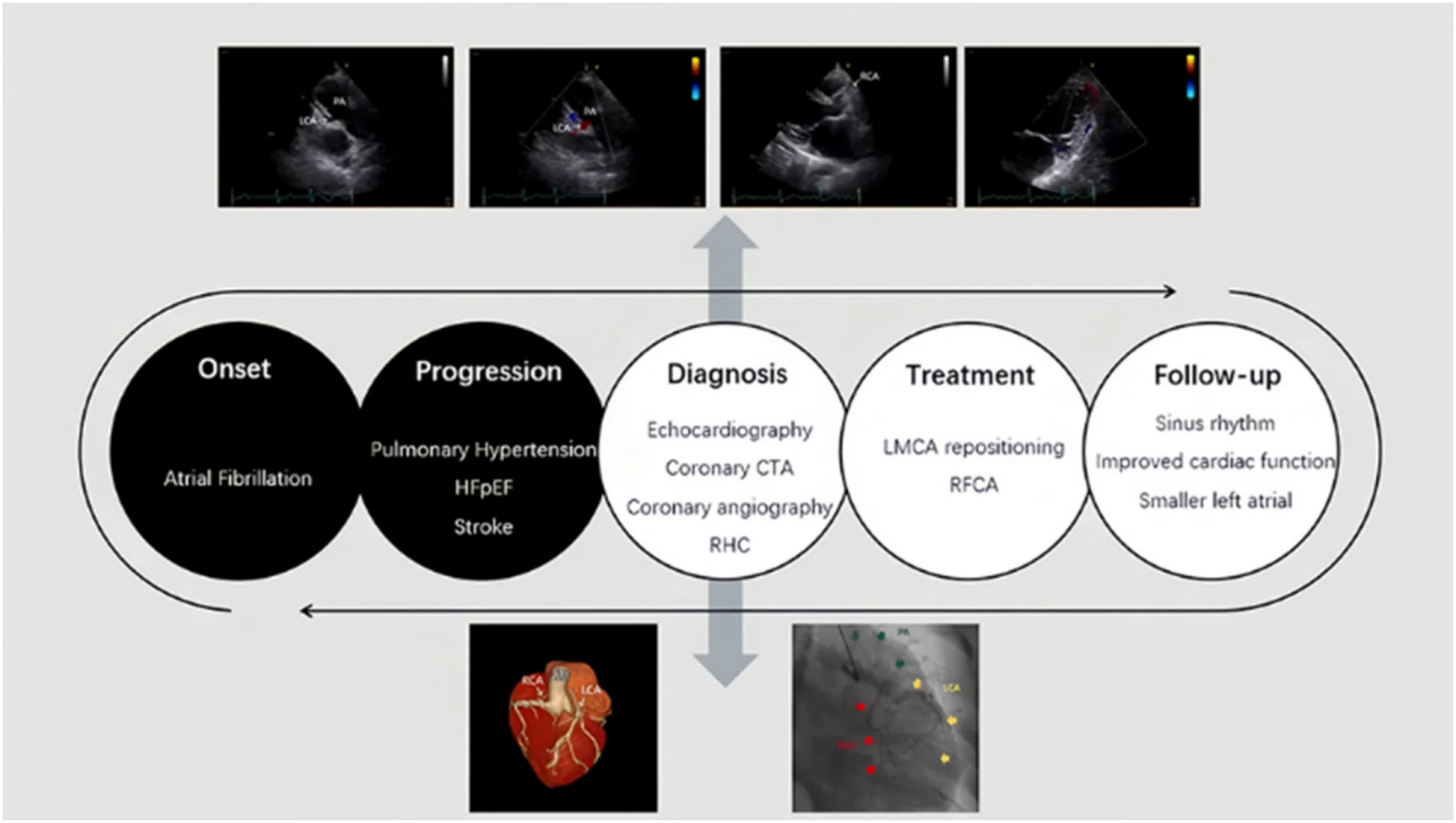
Timeline of the case. The timeline of the diagnosis and treatment of the patient.

A 28-year-old female was admitted to our hospital due to atrial fibrillation and pulmonary hypertension. She first experienced palpitations at the age of 20, and an ECG confirmed atrial fibrillation. Despite treatment with amiodarone, her condition escalated from paroxysmal to persistent atrial fibrillation. She has not undergone anticoagulation therapy. At the age of 27, her exercise tolerance notably declined, accompanied by chest pain post-activity, and suffered an ischemic stroke. The echocardiography estimated the pulmonary artery systolic pressure (PASP) at 67 mmHg. Although she was treated with oral ambrisentan, her exercise tolerance did not improve, leading her to seek further treatment at our institution. Her medical history was clear of hypertension, diabetes, hyperlipidemia, smoking, drug or food allergies, stimulant use, or any family history of cardiovascular disease. On physical examination, her vital signs were normal, with P2 > A2, irregular heart rhythm, and mild edema in both lower limbs. The patient's thyroid function, hepatic and renal function, high-sensitive troponin I were normal. The N-terminal pro-B-type natriuretic peptide (NT-proBNP) level was elevated at 2,180 pg/ml (normal range: 0–125 pg/ml). Her ECG showed atrial fibrillation with ST-segment depression in leads I, aVL and V4–V6, and poor R-wave progression in leads V1–V3 (Figure 1). Right heart catheterization (RHC) revealed the pulmonary artery systolic pressure, diastolic pressure, and mean pressure were 42 mmHg, 22 mmHg, and 30 mmHg, respectively. The pulmonary capillary wedge pressure (PCWP) was 23 mmHg, and the pulmonary vascular resistance (PVR) was measured at 1.26 Wood Units, suggesting the pulmonary hypertension was related to left heart disease (4). Her transthoracic echocardiography showed a left ventricular ejection fraction of 65%, with an enlarged left atrium with dimensions measuring 48 mm antero-posteriorly, 61 mm superior-inferiorly, and 50 mm transversely, and suspected origin of the LCA from the pulmonary artery. Additionally, the right coronary artery is dilated, and there is visible extensive blood flow communication between the left and right coronary arteries within the myocardium. Echocardiographic evaluation also revealed mild left ventricular enlargement, with left ventricular end-diastolic dimension 51.2 mm and left ventricular end-diastolic volume indexed 74.31 ml/m². Although no regional wall motion abnormalities were observed, the impaired diastolic function (E/e′ ratio 12) indicates chronic ischemia of the left ventricle. There are also signs of right heart dysfunction to chronic pulmonary hypertension with reduced tricuspid annular plane systolic excursion (TAPSE) 12 mm (Figure 2). Further coronary computed tomography angiography (CTA) and coronary angiography showed the left main coronary artery (LMCA) originating from the pulmonary artery, with multiple connections between the distal left and right coronary arteries, and a right coronary to left ventricle fistula (Figure 3 and Supplementary Video S1). Based on these observations, we thought that her clinical manifestations were due to anomalous coronary origin, specifically the abnormal origin of the left coronary artery from the pulmonary artery. The left atrium, mainly supplied by the left circumflex branch (LCX), suffered from prolonged ischemia, leading to structural and electrophysiological remodeling and subsequently to atrial fibrillation and heart failure with preserved ejection fraction (HFpEF), resulting in left heart failure-related pulmonary hypertension, and stroke. She underwent surgery intervention under general anesthesia with hypothermic cardiopulmonary bypass, including the LMCA repositioning and radiofrequency ablation for atrial fibrillation. Postoperative medications included warfarin and aspirin for thromboembolic prophylaxis, amiodarone for rhythm stabilization, and dapagliflozin for optimization in HFpEF phenotype. She was followed up six months after surgery. She regained sinus rhythm, demonstrated a significant improvement in exercise tolerance, and showed a decrease in NT-proBNP levels to 233 pg/ml. Echocardiography revealed a reduction in left atrial size to dimensions of 48 mm × 61 mm × 50 mm (antero-posterior × superior-inferior × transverse diameters) and a decreased estimated PASP from 60–47 mmHg. The timeline of the diagnosis and treatment of the patient is shown in Figure 4.

## Discussion

This article reports a case of an adult ALCAPA patient with atrial fibrillation and pulmonary hypertension due to left heart disease as clinical manifestations.

ALCAPA syndrome is classified into infant and adult types, each with different symptoms and outcomes (5). Infants typically suffer from myocardial infarction and congestive heart failure, with approximately 90% dying within the first year. In adults, ALCAPA is less common. Patients may present with symptoms of angina pectoris, dyspnea, syncope, and palpitations, and it can be a significant factor in sudden cardiac death (3, 6). When presented in adulthood, ALCAPA typically shows abundant interarterial collateral vessels between the RCA and the LCA (7). This was confirmed by coronary angiography and coronary CTA in this case and is the reason why she did not die at an early age.

Yau et al. summarize the clinical manifestations of adult patients with ALCAPA, noting that 66% of cases present with symptoms such as angina, dyspnea, palpitations, or fatigue; 17% with ventricular arrhythmia, syncope, or sudden death; and 14% remain asymptomatic (8). However, previous studies have not clearly defined the proportion of adult ALCAPA patients presenting with atrial fibrillation or pulmonary hypertension as clinical manifestations. In this case, we observe a typical presentation with reduced exercise tolerance and exertional angina, which is consistent with documented manifestations in the literature (1, 5, 8). While cardiopulmonary exercise testing was not obtained, her exertional symptoms (NYHA Class II-III), ischemic stroke, and objective hemodynamic derangements collectively indicated significant functional impairment. However, when the patient first presented to us, her more prominent clinical manifestations were atrial fibrillation and pulmonary hypertension. She was treated with amiodarone and ambrisentan, but the therapeutic effects were poor. Echocardiography showed that her left ventricular systolic function is normal with mild mitral regurgitation and impairment in diastolic function, while the left atrium was significantly enlarged. The results of the RHC confirmed that the patient's pulmonary hypertension was left heart-related. We hypothesized that the cause was due to the origin of the LMCA in the pulmonary artery, resulting in prolonged ischemia in the left atrium, which is mainly supplied by the LCX (9). These collaterals between the LCA and RCA, although beneficial for blood supply from the LCA, do not completely alleviate the myocardial ischemia caused by the anomalous origin of the coronary artery. Long-term ischemia leads to left atrial enlargement, contributing to structural and electrical remodeling of the atrium, which plays a significant role in the development of atrial fibrillation (10). The patient also exhibited chronic left ventricular (LV) ischemia evidenced by ECG changes, LV dilation and diastolic dysfunction, and elevated NT-proBNP. While collateral circulation prevented regional wall motion abnormalities and reduced ejection fraction, the above manifestations are still important evidence of left ventricular ischemia. The pulmonary hypertension in this patient resulted from left heart disease, driven by ischemic injury to both the left atrium and left ventricle. While atrial fibrillation initially manifested at age 20—likely triggered by early left atrial hypertension from this dual ischemia—the overt pulmonary hypertension developed later as sustained left atrial overload progressively impacted the pulmonary circulation. As a result, we discontinued the use of ambrisentan. The development of pulmonary hypertension in ALCAPA may confer a paradoxical protective effect by reducing the coronary steal pressure gradient. While our patient's post-capillary PH likely had limited hemodynamic impact, this mechanism could explain her preserved systolic function despite chronic ischemia, underscoring the complex interplay between pulmonary and coronary physiology in shunt lesions.

In patients with postnatal ALCAPA, the direction of blood flow is usually left-to-right shunting, which has been repeatedly demonstrated (1, 11). Initially, both the LCA and the RCA have normal antegrade flow, because the pulmonary arterial pressure is equal to the systemic pressure (12). However, shortly after birth, the decrease in pulmonary artery pressure causes a reversal of flow in the LCA, causing the LCA to drain oxygenated blood into the lower pressure pulmonary artery. This creates a preferential flow toward the pulmonary circulation rather than the myocardial circulation, which manifests as a left-to-right shunt known as the steal phenomenon (2, 3). This was also verified by the finding of the blood flow from LCA to the pulmonary artery on echocardiography examination (Figure 2b2).

Surgical intervention remains the cornerstone of treatment for ALCAPA to restore normal coronary anatomy and alleviate symptoms (5, 6, 13). The patient underwent the left coronary artery reimplantation and radiofrequency ablation for atrial fibrillation. These procedures significantly improved her clinical outcome.

## Conclusion

This case highlights the importance of considering ALCAPA in the differential diagnosis of young patients presenting with atrial fibrillation and pulmonary hypertension.

### Patient perspective

After years of unexplained cardiac symptoms, being diagnosed with the rare congenital heart defect of Anomalous Origin of the Left Coronary Artery from the Pulmonary Artery was lifesaving for me. The care and expertise at Peking Union Medical College Hospital were pivotal in accurately diagnosing and effectively treating my condition. I am immensely grateful to the dedicated team of cardiologists whose efforts have significantly improved my quality of life.

## Data Availability

The original contributions presented in the study are included in the article/Supplementary Material, further inquiries can be directed to the corresponding authors.

## Supplementary material

Supplementary Video S1 *(as Listing Supplementary Material for Review).* Coronary angiography showed that after the injection of contrast medium, the right coronary artery, left coronary artery, and pulmonary artery were sequentially highlighted. Extensive communication between the left and right coronary arteries was also observed.
